# Influence of Polymers, Cobalt Oxide Nanoparticles, and Organophilized Palygorskite on Heat Storage and Filtrate Volume of Water-Based Drilling Fluid

**DOI:** 10.1021/acsomega.6c04516

**Published:** 2026-09-09

**Authors:** Iane Barbosa Augusto Galvão, Pedro Lucas Araújo da Silva, Patrícia Mendonça Pimentel, Rhaul Phillypi da Silva, Alcides de Oliveira Wanderley Neto, Marcos Allyson Felipe Rodrigues, Edney Rafael V. P. Galvão, Vanessa C. Santanna

**Affiliations:** † Graduate Program in Petroleum Engineering, 28123 Federal University of Rio Grande do Norte, Natal, Rio Grande do Norte CEP 59072-970, Brazil; ‡ Department of Exact Sciences and Information Technology, Federal Rural University of Semi-Arid, Mossoró, Rio Grande do Norte CEP 59625-900, Brazil; § Graduate Program in Petroleum Engineering, Federal University of Sergipe, São Cristóvão, Sergipe CEP 49107-230, Brazil; ∥ Institute of Chemistry, Federal University of Rio Grande do Norte, Natal, Rio Grande do Norte CEP 59072-970, Brazil

## Abstract

The application of nanofluids has gained prominence in the oil industry, particularly in the improvement of drilling fluids. The incorporation of metal oxide nanoparticles, such as cobalt oxide, contributes to enhanced thermal, rheological, and fluid-loss control properties due to their greater thermal and chemical stability. Palygorskite clay stands out for its ability to act as a viscosifier in saline environments, forming stable suspensions. However, organophilized palygorskite may contribute to the formation of a low-permeability filter cake in water-based fluids. In this context, this study evaluates the effects of the interaction between polymers, cobalt oxide nanoparticles, and organophilized palygorskite clay in a water-based drilling fluid. The rheological properties, static filtration, and thermal performance of fluids containing cobalt oxide nanoparticles and organophilized palygorskite were investigated at different concentrations and temperatures ranging from 25 to 70 °C. The results showed that fluids containing organophilized palygorskite exhibited lower filtrate volume, while fluids containing cobalt oxide nanoparticles improved heat retention, highlighting their potential for use at lower temperatures.

## Graphical abstract

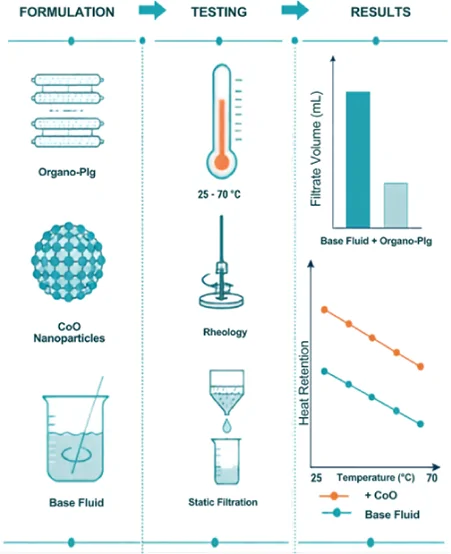

## 1 Introduction

Drilling fluid is essential for enabling the oil extraction process, performing several operational functions. Among these, the removal of drill cuttings, maintenance of hydrostatic pressure inside the well, lubrication of the drill bit, and formation of a low-permeability filter cake on the wall of the drilled geological formation stand out.^1^ In this context, the incorporation of nanoparticles into drilling fluids has been widely studied as a strategy to improve their thermal, rheological, and filtration properties.^2–9^

Paixão et al.^10^ investigated how the use of nanoparticles can contribute to increasing heat storage capacity, reducing cooling effects on the rheological properties of water-based drilling fluids. For this purpose, a hybrid material composed of magnetic iron, silica, and silver nanoparticles was developed. It was observed that the presence of Fe_3_O_4_@SiO_2_@Ag nanoparticles resulted in decreased viscosity, yield stress, and gel strength. In addition, they found that nanoparticles can be used as a heat exchange medium, resulting in fluids with improved rheological properties at low temperatures. Similarly, Ahmed et al.^11^ synthesized hybrid SiO_2_/g-C_3_N_4_ nanoparticles for use in water-based drilling fluids. The results indicated significant improvements in yield point and 10 s gel strength. Filtrate volume was reduced by 69.6 to 88.7% when 0.5 to 1.5 lb/bbl of nanoparticles were used in the formulations. In another study, Abdullah et al.^12^ synthesized a graphene oxide–polyethylenimine nanocomposite (PEI-GO). They reported that 0.3 wt % of this additive increased plastic viscosity and yield point, resulting in up to a 67% reduction in filtrate volume under conditions of 160 °C and high salinity. Furthermore, Dai and He^13^ used zinc oxide (ZnO) nanocomposites with polyacrylamide (PAM) as an additive to enhance the performance of water-based drilling fluids. The incorporation of the nanocomposite at concentrations between 0.1 and 1.0% significantly improved performance parameters, with an increase in apparent viscosity from 35 cP to 48 cP, as well as a reduction in API filtrate volume from 12.8 mL to 7.2 mL and from 24.5 mL to 15.8 mL in HTHP tests at 150 °C.

In addition to nanoparticles, the selection of natural additives such as organophilized palygorskite clay also plays a crucial role in the formulation of drilling fluids, as it can act by forming a filter cake impermeable to the aqueous phase of the fluid, thereby reducing filtrate. Zhang et al.^14^ evaluated the colloidal stability, rheological properties, and filtration behavior of mixed palygorskite–montmorillonite clays in freshwater and saltwater systems. The results showed that palygorskite exhibits good rheological performance in both freshwater and saltwater, while montmorillonite performs better in freshwater. The addition of palygorskite improved colloidal stability and rheological properties of mixed clays in seawater. Likewise, Gonçalves et al.^15^ analyzed palygorskite in saline water-based fluids, especially as a rheological agent, using different concentrations of palygorskite and low-viscosity polyanionic cellulose (PAC LV). These formulations were evaluated through HPHT filtrate volume measurements, rheological properties, density, and pH. The results indicated that the presence of palygorskite promotes up to a 21% reduction in filtrate volume.

Based on the literature on the use of nanoparticles and clays in drilling fluids, few studies have addressed the thermal analysis of these systems. Typically, only rheology and filtration are evaluated. Therefore, this work is innovative in demonstrating how the interaction between polymers/cobalt oxide nanoparticles and polymers/organophilized palygorskite clay influences thermal energy storage and filtrate volume in water-based fluids.

## 2 Materials and Methods

### 2.1 Preparation of Nanoparticles

Cobalt oxide nanoparticles (CoO NPs) were synthesized using the gelatin method reported by Oliveira et al.^16^ Gelatin powder (GELITA) and cobalt nitrate hexahydrate (Co­(NO_3_)_2_·6H_2_O, 99.9%, Atriom) were used as starting materials. Initially, gelatin powder was dissolved in 150 mL of deionized water under constant stirring at 50 °C until complete dissolution. Subsequently, a stoichiometric amount of cobalt nitrate was added to the solution under stirring at 70 °C for 30 min. The temperature was then gradually increased to 90 °C until a viscous resin was formed. The resulting resin was calcined at 900 °C for 4 h with a heating rate of 10 °C min^–1^ to obtain the CoO nanoparticles.

### 2.2 Characterization of the Nanoparticles

The synthesized NPs were characterized using various techniques. The structure of the NPs was determined using X-ray diffraction (XRD) analysis. The XRD traces were recorded on an X-ray diffractometer (7000 Shimadzu) using Cu-*K*α radiation with a wavelength of 1.5406 Å. Crystallite sizes were obtained using Scherrer’s equation. This equation is used to estimate the average crystallite size from the broadening of XRD peaks.^17^

Morphological analysis of the powders was performed using a field emission gun scanning electron microscope (FEG-SEM; TM3000 Hitachi). The Brunauer–Emmett–Teller (BET) and Barrett–Joyner–Halenda (BJH) methods were used to determine the specific surface area and pore size, respectively. These measures were obtained using a NOVA2000 BET system.

### 2.3 Preparation and Characterization of Organo-Palygorskite

In this work, 3.4 wt % of palygorskite (Plg) was added to 6.5 g L^–1^ cetyltrimethylammonium bromide (CTAB) and stirred at 200 rpm on a shaking table for 1 h at 25 °C.^18^ After a subsequent 24 h resting period, supernatants were removed, and the Plg was dried in an oven at 60 °C for 24 h, obtaining organo-palygorskite (organo-Plg). Mineralogical composition and the thermal curves of organo-Plg can be seen in the work of Silva *et al*.^18^ The X-ray diffraction (XRD) patterns and scanning electron microscopy (SEM) images of the organo-Plg were obtained using the same equipment employed for the cobalt oxide characterization.

### 2.4 Drilling Fluid Preparation

Five aqueous drilling fluids were prepared (in duplicate). A fluid without nanoparticles and without clay, considered a standard fluid for comparison; two fluids containing cobalt oxide nanoparticles (CoO NPs) at concentrations of 0.25 and 0.5 wt %; and two fluids containing organophilized palygorskite clay (organo-Plg) at concentrations of 0.25 and 0.5 wt %. These concentrations were chosen, as they are within the range commonly reported in the literature (up to 3 wt %).^10,12,13,19–24^ Table 1 shows the functions and concentrations of all of the chemicals used in the preparation of this drilling fluid. This fluid formulation was developed based on those commonly used in offshore sandstone reservoirs in Brazil.

**Table 1:** Composition of the Aqueous Drilling Fluid

Component	Function	Quantity
Fresh water	Dispersing medium	471 mL
Xanthan gum	Viscosity enhancer	1.43 g
Hydroxypropylamine (HPA)	Filtrate reducer	8.58 g
Low-viscosity polyanionic cellulose (PAC LV)	Filtrate reducer	2.86 g
Sodium chloride (NaCl)	Clay inhibitor	22.60 g
Triazine	Bactericide	0.43 g
Calcium carbonate	Sealing material	28.60 g

For the preparation of the fluids, distilled water, xanthan gum, HPA, and PAC LV were added to a Hamilton Beach mixer. The mixing time was 10 min for each polymer. Subsequently, NaCl, triazine, and calcium carbonate were added, with a mixing time of 5 min for each additive. In the fluids containing CoO NPs and clay, both were added after the xanthan gum. After preparation, the fluids were characterized by carrying out analyses in duplicate.

### 2.5 Characterization of the Drilling Fluids

The parameters of the prepared drilling fluid samples were measured following the specifications and standard procedures of API RP 13B-1.^25^ The rheological parameters were measured using an OFITE rotational viscometer (model 800) at 25, 50 and 70 °C. Apparent viscosity (AV), plastic viscosity (PV), yield point (YP) and gel strength (GS) were calculated according to eqs 1–4:

1 AV(cP)=L600/2

2 PV(cP)=L600−L300

where L600 = dial reading at 600 rpm of the rotational viscometer and L300 = dial reading at 300 rpm of the rotational viscometer.

3 $$\mathrm{YP}\left({\mathrm{lbf}\;100\;\mathrm{ft}}^{-2}\right)=L300-\mathrm{PV}$$

4 $$\mathrm{GS}\left({\mathrm{lbf}\;100\;\mathrm{ft}}^{-2}\right)=G_{\mathrm{final}}{-G}_{\mathrm{initial}}$$

Where lbf denotes pounds-force; G_initial_ and G_final_ are the initial and final gel strengths, respectively, measured after 10 s and 10 min under static conditions and determined from the maximum dial reading obtained at 3 rpm. GS represents the degree of thixotropy of the drilling fluid.

An OFITE HPHT press-filter was used to determine the fluid filtrate volume (FV), fluid loss coefficient (C_w_) and spurt volume (V_SP_). A pressure of 100 psi was applied at 25 °C and 300 psi at 50 and 70 °C, and the test period began at the time of pressure application. The volume of filtrate collected was measured at 1, 2, 3, 4, 5, 10, 15, 20, 25, and 30 min. Graphs of FV per filtering area unit were plotted as a function of the square root of time. C_w_ was estimated from the slope of the fitted straight line, and V_SP_ was determined from the linear coefficient of the equation of the straight line.

Analysis of the fluid thermal performance was carried out by initially placing 100 g of the fluid at 26 °C in a water bath at 60 °C. The fluid was heated until it reached a temperature of 60 °C, and the temperature was monitored every minute. The fluid was then transferred to an ultrathermostatic bath at 8 °C to cool down until it reached a temperature of 8 °C. Again, the temperature was monitored every minute. A graph of temperature as a function of time was then plotted for each fluid. Considering the water bath and the ultrathermostatic bath as hot and cold thermal reservoirs, respectively, it is possible to use Newton’s law differential equation of heating and cooling to estimate the heating (κ_h_) and cooling (κ_c_) coefficients, which predict an exponential increase (heating) or decrease (cooling) in temperature over time, as described by eqs 5 and 6,^26,27^ respectively:

5 $$T\left(t\right)=T_{\mathrm{water}}-\left(T_{\mathrm{water}}{-T}_{0}\right)\times \exp^{-\kappa_{h}t}$$

Where: T­(t) is the fluid temperature at time t; T_0_ is the initial fluid temperature (26°C); T_water_ is the water bath temperature (60°C).

6 $$T\left(t\right)=T_{\mathrm{water}}+\left(T_{0}{-T}_{\mathrm{water}}\right)\times \exp^{-\kappa_{c}t}$$

Where: T­(t) is the fluid temperature at time t; T_0_ is the initial fluid temperature (60°C); T_water_ is the water bath temperature (8°C).

## 3 Results and Discussion

### 3.1 Characterization of the Nanoparticles and Organo-Palygorskite

The X-ray diffraction pattern of the synthesized CoO sample is presented in Figure 1. The diffraction peaks observed were indexed to the planes of cubic CoO (a = 4.261 Å, spatial group Fm3̅m), consistent with the JCPDS card (48-1719), confirming the formation of the crystalline CoO phase. However, additional diffraction peaks of the spinel Co_3_O_4_ phase were also observed. The relatively sharp diffraction peaks suggest a high degree of crystallinity of the synthesized material. The average crystallite size was estimated using Scherrer’s equation, resulting in a value of approximately 53.6 nm, confirming the nanostructured nature of the sample.

**Figure 1:**
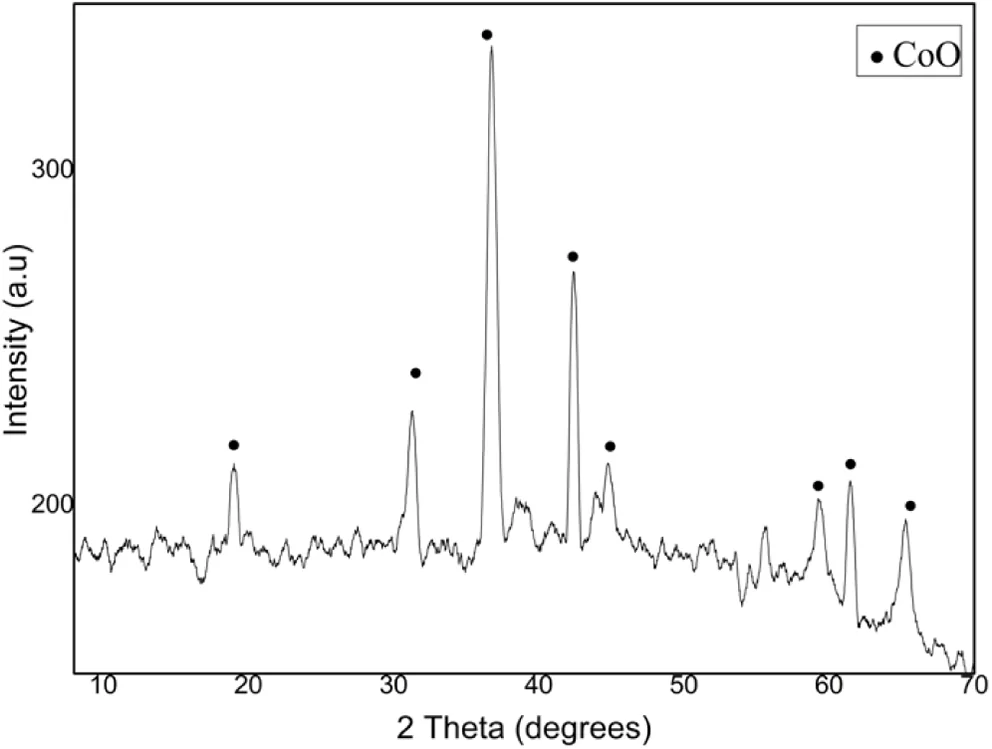
XRD trace of CoO.

The X-ray diffractogram of organo-Plg (Figure 2) shows that the sample retained the characteristic crystalline structure of palygorskite after organic modification, indicating that the treatment did not significantly alter the clay framework. Nevertheless, a considerable amount of quartz and minor traces of kaolinite were detected. The coexistence of these mineral phases is commonly reported in natural palygorskite deposits.^28–30^

**Figure 2:**
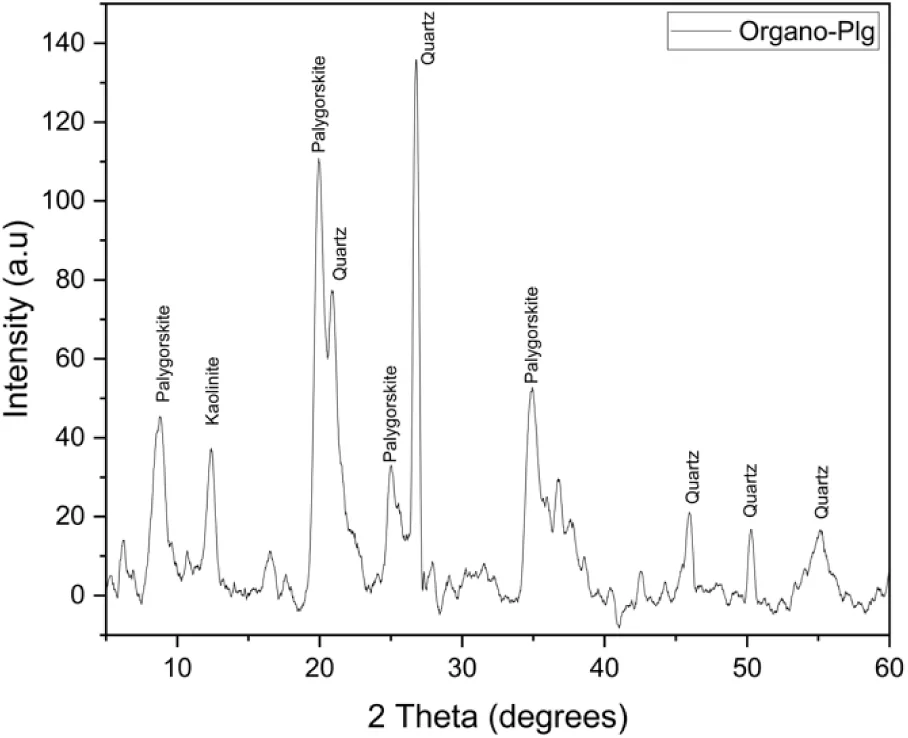
XRD trace of organo-Plg.

The SEM image of CoO NPs (Figure 3) reveals highly agglomerated particles with a porous structure. The observed agglomeration is typical of oxide nanoparticles synthesized by polymeric precursor routes, since the nanometric particle size leads to high surface energy, promoting particle clustering. As a consequence, weakly bound agglomerates are formed due to the presence of weak van der Waals forces, making them easily deagglomerated.^16,31,32^ The porosity of the sample results from the release of gases generated during the decomposition of gelatin throughout the calcination process. The average particle size, as determined by comparison with the scale bar, was below 70 nm, confirming the nanostructured nature of the CoO.

**Figure 3:**
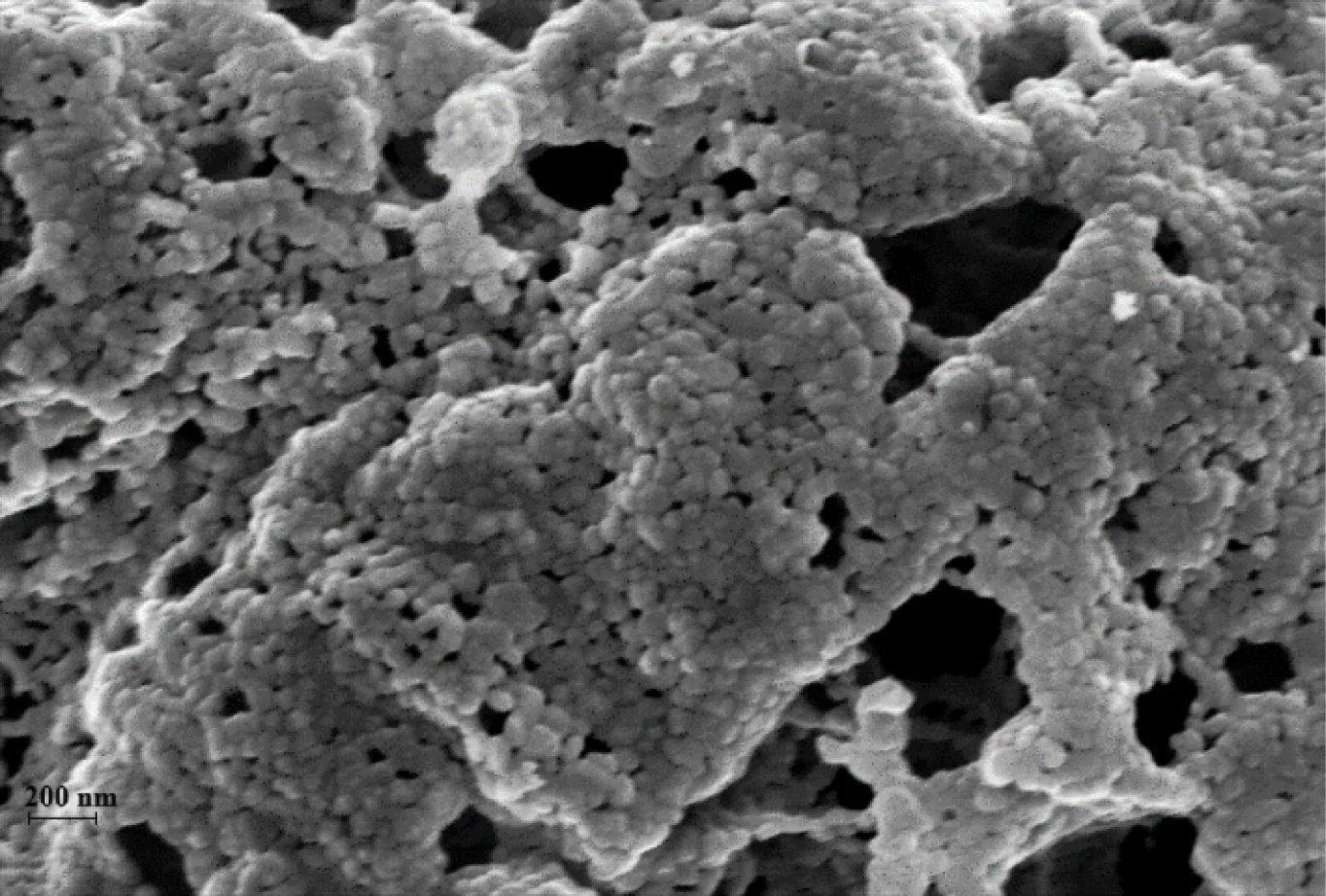
SEM image of CoO NPs under 30000× magnification.

Figure 4 presents the SEM image of the organo-Plg. The material exhibits a fibrous morphology characterized by flat, straight fibers randomly oriented and entangled into bundles. In addition, particle agglomeration and partial surface coverage were observed, which may be associated with the adsorption of organic species onto the clay surface.

**Figure 4:**
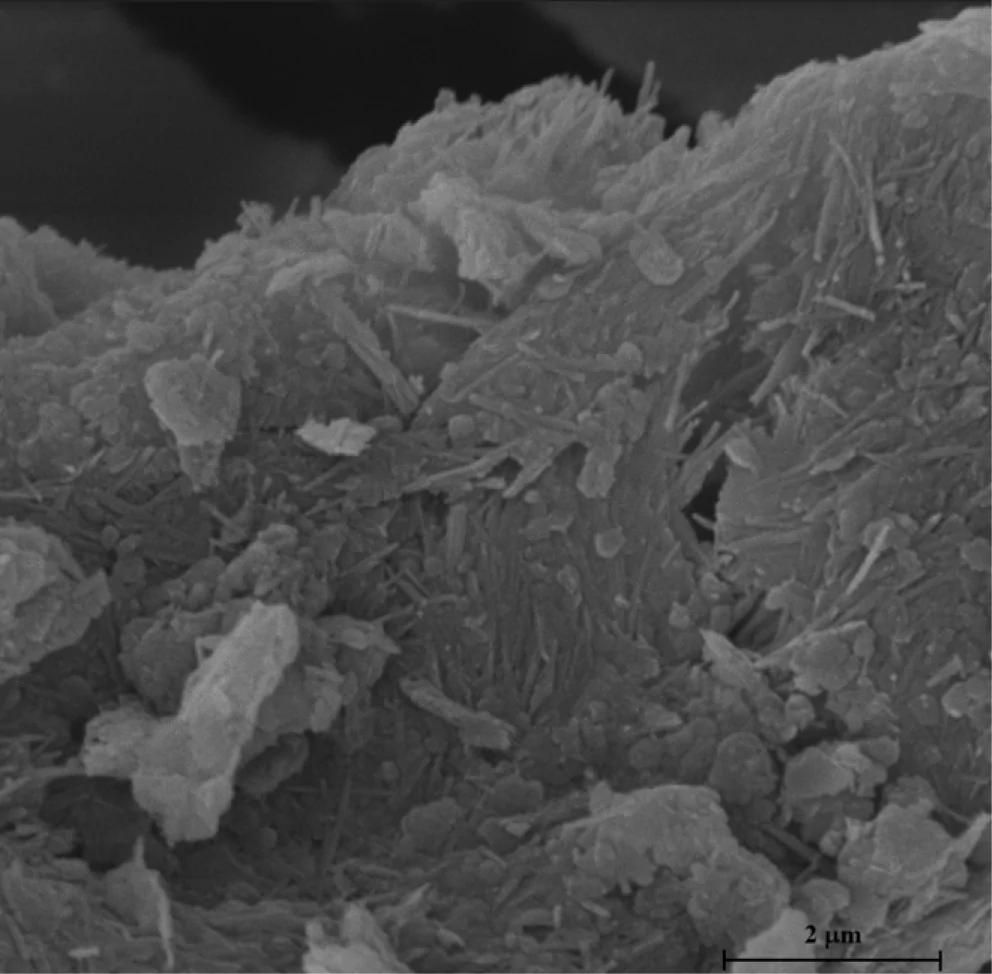
SEM image of organo-Plg under 30000× magnification.

### 3.2 Rheological Analysis of the Fluids

Flow curves (shear stress versus shear rate) were obtained for the studied fluids at temperatures ranging from 25 to 70 °C (Figure 5). As illustrated in Figure 5, all formulations exhibited pseudoplastic behavior with a yield stress, a rheological characteristic commonly observed in drilling fluids. The flow curves remained closely grouped over the investigated temperature range, suggesting that the fluids experienced only a slight decrease in flow resistance as the temperature increased.

**Figure 5:**
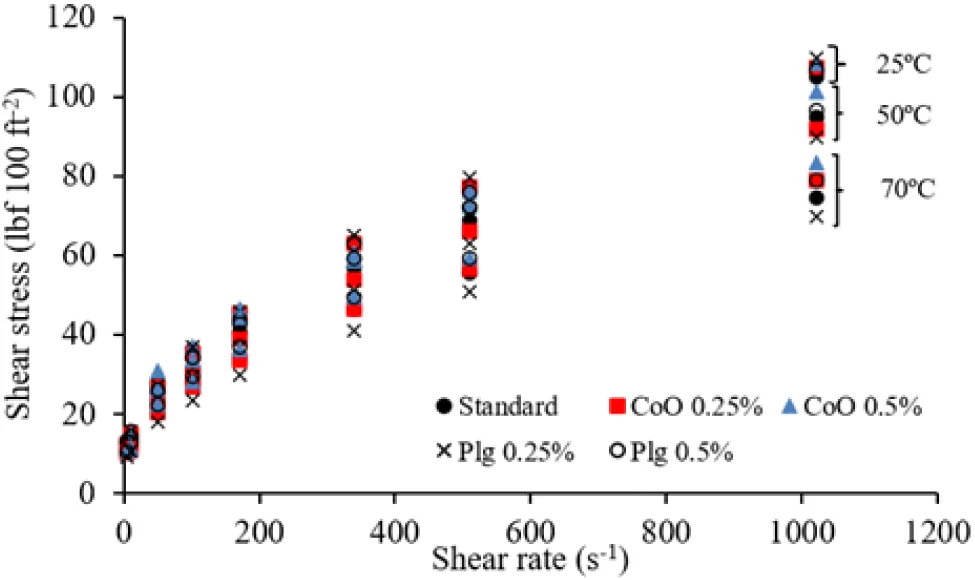
Dynamic flow behavior of fluids 25–70 °C.

The average values of apparent viscosity (AV), plastic viscosity (PV), yield point (YP), and gel strength (GS), along with the respective standard deviations of the dataset, are presented in Table 2.

**Table 2:** Properties of the Drilling Fluids^a^

Property	Standard fluid	CoO 0.25%	CoO 0.5%	Plg 0.25%	Plg 0.5%
25 °C					
AV/cP	49.3 (±0.8)	50.3 (±0.8)	50.8 (±0.8)	51.5 (±0.8)	50.0 (±0.8)
PV/cP	26.0 (±0.8)	28.0 (±0.8)	30.0 (±0.8)	28.5 (±0.8)	29.0 (±0.8)
YP/(lbf/100 ft^2^)	46.5 (±1.7)	44.5 (±1.7)	41.5 (±1.7)	46.0 (±1.7)	42.0 (±1.7)
GS/(lbf/100 ft^2^)	2.5 (±0.6)	3.0 (±0.6)	2.0 (±0.6)	2.5 (±0.6)	2.5 (±0.6)
50 °C					
AV/cP	44.5 (±0.8)	43.0 (±0.8)	47.5 (±0.8)	42.0 (±0.8)	45.3 (±0.8)
PV/cP	24.5 (±1.1)	24.0 (±1.1)	27.0 (±1.1)	25.0 (±1.1)	23.0 (±1.1)
YP/(lbf/100 ft^2^)	40.0 (±1.5)	38.0 (±1.5)	41.0 (±1.5)	34.0 (±1.5)	44.5 (±1.5)
GS/(lbf/100 ft^2^)	1.5 (±0.5)	1.5 (±0.5)	3.0 (±0.5)	2.0 (±0.5)	2.0 (±0.5)
70 °C					
AV/cP	35.0 (±1.4)	37.0 (±1.4)	39.0 (±1.4)	32.8 (±1.4)	37.0 (±1.4)
PV/cP	18.0 (±1.5)	21.0 (±1.5)	22.5 (±1.5)	18.0 (±1.5)	18.5 (±1.5)
YP/(lbf/100 ft^2^)	34.0 (±0.8)	32.0 (±0.8)	33.0 (±0.8)	29.5 (±0.8)	37.0 (±0.8)
GS/(lbf/100 ft^2^)	0.0 (±0.4)	1.0 (±0.4)	1.0 (±0.4)	1.0 (±0.4)	1.5 (±0.4)

a Mean and standard deviation of the set of estimates (in parentheses).

At 25 °C, the results for apparent viscosity, yield point, and gel strength, considering the obtained standard deviation (Table 2), indicate that the presence of CoO NPs and organo-Plg did not influence these fluid properties. Regarding plastic viscosity, the results show a slight increase in PV with the addition of CoO NPs and organo-Plg compared to the standard fluid, which is expected since PV increases with increasing solids concentration in the fluid. The greater plastic viscosity results from the flocculation and aggregation of NPs and organo-Plg, which may cause greater attraction, poorer distribution, and a reduced distance between the solids.^9^

At 50 °C, a reduction in apparent viscosity and plastic viscosity was observed for all fluids compared to 25 °C (Table 2), which is expected behavior. However, the presence of CoO NPs and organo-Plg did not produce a significant effect on these properties. Regarding yield point, organo-Plg at 0.5 wt % led to an increase in this parameter, indicating stronger electrochemical interactions within the fluid.^33^ YP is an indicator of the strength of the fluid microstructure under dynamic flow conditions.^33^ Accordingly, the fluid formulated with 0.5 wt % organo-Plg exhibited higher flow resistance, which may enhance the dynamic suspension capacity of drill cuttings and improve borehole cleaning efficiency during drilling operations.^34^ As for gel strength, the fluid containing 0.5 wt % CoO NPs remained slightly more stable at this temperature, considering the standard deviation (Table 2), which is an important factor for maintaining cuttings suspension during circulation shutdown.

At 70 °C, a further reduction in apparent viscosity and plastic viscosity was observed for all fluids (Table 2) compared to 50 °C, as expected. Again, the presence of CoO NPs and organo-Plg did not significantly affect these properties. Regarding yield point, organo-Plg at 0.5 wt %, as observed at 50 °C, resulted in an increase in this parameter, indicating that electrochemical interactions remain strong even with increasing temperature. As for gel strength at this temperature, no significant differences were observed among fluids containing CoO NPs and organo-Plg. However, the standard fluid did not exhibit thixotropy at this temperature, showing a gel strength equal to zero. Organo-Plg particles may exhibit heterogeneous surface charge distribution, including localized positively charged sites.^18^ CoO nanoparticles can present Lewis’s acid–base surface characteristics due to exposed cobalt and oxygen sites, enabling interactions with polymer chains present in the fluid. These interactions may promote the formation of a three-dimensional network structure, contributing to gel formation and the thixotropic behavior of the suspension.^33^

### 3.3 Static Filtration

During the HPHT filtration tests, the filtrate volume was obtained for each fluid studied. Figures 6–8 present the cumulative filtrate volume as a function of the square root of time for the five fluids at the three predefined temperatures. From the linear equations obtained for each fluid, the slope corresponds to the fluid loss coefficient (C_w_), while the intercept represents the spurt loss. The values of C_w_, along with the filtrate volumes, are presented in Table 3.

**Figure 6:**
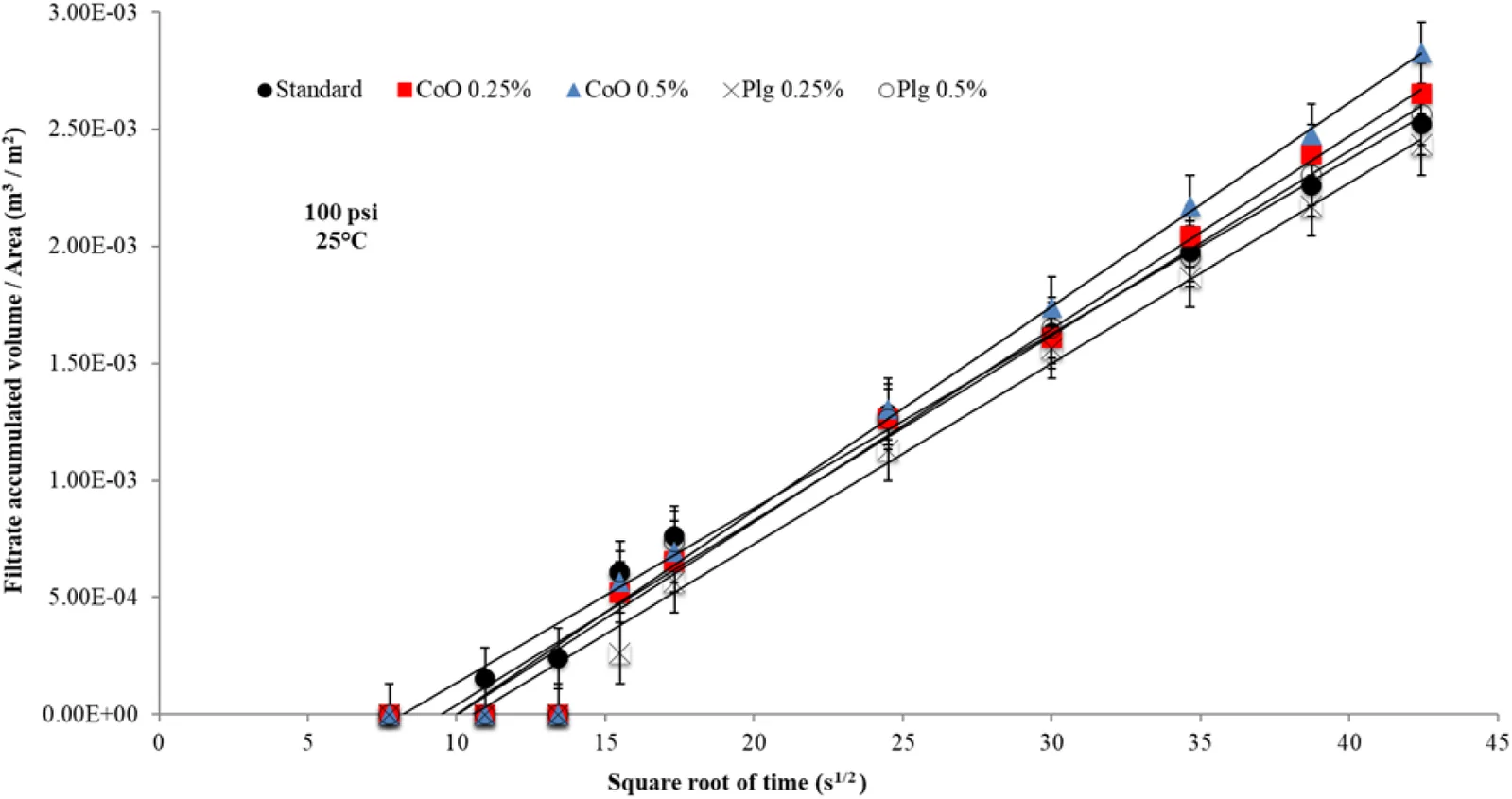
Accumulated filtrate volume as a function of the square root of time for all investigated drilling fluids at 25 °C and 100 psi.

**7:**
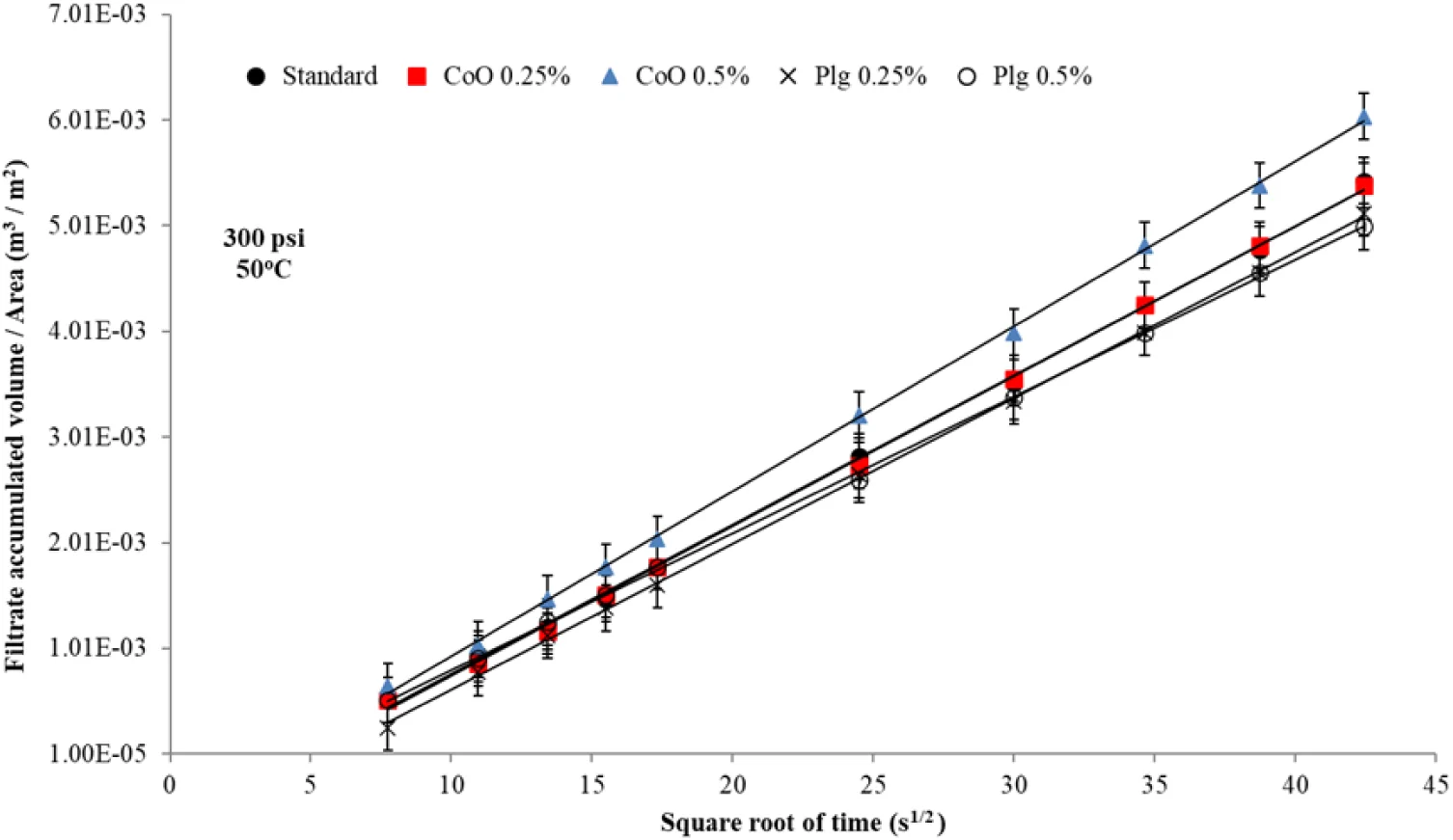
Accumulated filtrate volume as a function of the square root of time for all investigated drilling fluids at 50 °C and 300 psi.

**Figure 8:**
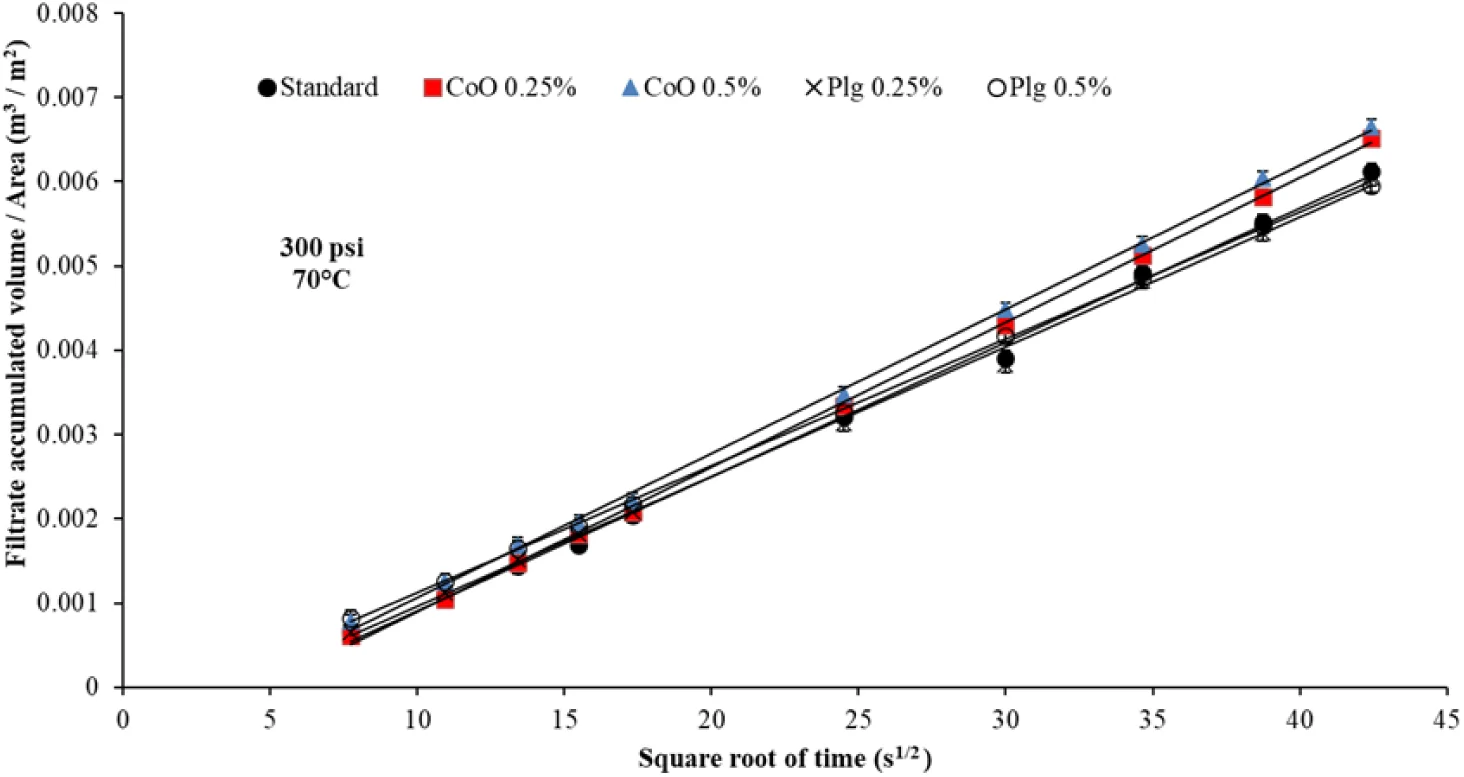
Accumulated filtrate volume as a function of the square root of time for all investigated drilling fluids at 70 °C and 300 psi.

**Table 3:** Filtration Parameters of the Studied Drilling Fluids

Fluid at 25 °C	C_W_ (m/s^1/2^)	Filtrate volume (mL)	Standard deviation
Standard	7 × 10^–5^	5.8	±0.3
CoO 0.25%	8 × 10^–5^	6.1	±0.3
CoO 0.5%	9 × 10^–5^	6.5	±0.3
Plg 0.25%	7 × 10^–5^	5.5	±0.3
Plg 0.5%	8 × 10^–5^	5.9	±0.3
Fluid at 50 °C			
Standard	1 × 10^–4^	12.5	±0.5
CoO 0.25%	1 × 10^–4^	12.4	±0.5
CoO 0.5%	2 × 10^–4^	13.9	±0.5
Plg 0.25%	1 × 10^–4^	11.8	±0.5
Plg 0.5%	1 × 10^–4^	11.5	±0.5
Fluid at 70 °C			
Standard	2 × 10^–4^	14.1	±0.2
CoO 0.25%	2 × 10^–4^	15.0	±0.2
CoO 0.5%	2 × 10^–4^	15.2	±0.2
Plg 0.25%	2 × 10^–4^	13.9	±0.2
Plg 0.5%	2 × 10^–4^	13.7	±0.2

After analyzing Figures 6 to 8, it is observed that all fluids, at the three temperatures, exhibited negative spurt loss values. This indicates that the pores of the filter paper used in the test to determine filtrate volume were instantaneously plugged by the fine solids present in the fluids when pressure was applied. Negative spurt loss values have no physical meaning and confirm the null filtrate volume experimentally obtained at the initial moment of filtration.^35,36^

According to Table 3, the fluid containing organo-Plg (0.5 wt %) showed a reduction in filtrate volume at 50 °C compared to the standard fluid, considering the obtained standard deviation. This behavior may be associated with the clay’s ability to form a more compact and less permeable filter cake, enhancing liquid phase retention. The higher YP exhibited by this fluid at 50 °C may also account for its lower FV, as the increased structural strength of the fluid enhances its resistance to flow. However, at 70 °C, this effect was not observed, suggesting that the temperature increase intensified organo-Plg aggregation, also driven by its interaction mechanism with the PAC LV polymer. According to Yang et al.,^37^ PAC LV (anionic) adsorbs onto suspended clay particles, neutralizing the positive charges of organo-Plg. This increases electrostatic attraction between particles, promoting aggregation. As a result, the amount of free water available increases, leading to higher filtrate volume. In the work developed by Gonçalves et al.,^15^ the opposite behavior was observed, since palygorskite was used *in natura*, without organophilization, thus presenting a negatively charged surface.

For fluids containing CoO NPs, an increase in filtrate volume was observed at all evaluated temperatures. This behavior may be related to flocculation of the polymers present in the CoO NPs fluid. According to Mai et al.,^38^ the main mechanism of flocculation of fine suspended particles is through polymer bridging, where polymer molecules are absorbed on to the particles surface. As temperature increases, the polymer becomes more hydrophobic, resulting in aggregation of the polymer-metal oxide particles, forming so-called flocs. Salt also plays an important role in the flocculation of metal oxide particles. In the experiments, flocculation can also be evidenced by the higher gel strength observed for the fluid containing 0.5 wt % CoO at 50 °C (Table 2). This flocculation leads to the formation of a more porous and permeable filter cake, increasing the filtrate volume.

The fluid loss coefficient (C_w_) increased with temperature for all fluids, indicating lower resistance to filtration under higher thermal conditions, which is consistent with increased mobility of the liquid phase, possible changes in polymer–clay and polymer–NPs interactions, which may disrupt the colloidal network structure of the fluids. The increase in C_w_ observed for the CoO NPs fluid suggests that the nanoparticles promoted greater deposition of solids on the filter medium, resulting in a thicker, more heterogeneous, and more permeable filter cake, as evidenced by the simultaneous increase in FV. As discussed in the previous section, this behavior may be attributed to the aggregation of polymer–metal oxide NPs. The organo-Plg fluid exhibited the same C_w_ value as the standard fluid; however, it reduced the FV at 50 °C. This result is likely associated with the formation of a thinner, more compact, and less permeable filter cake. According to Evaristo et al.,^9^ filtration losses largely depend on the concentration and particle size of bridging agents, such as clays. Therefore, the organo-Plg microparticles likely acted as effective bridging agents within the porous medium, reducing the filtrate volume by improving filter-cake quality and sealing efficiency.

### 3.4 Thermal Performance of the Fluids

In the thermal performance test, the influence of CoO NPs and organo-Plg on the heating and cooling rates (heat flow) of water-based drilling fluid was evaluated. A comparative study was conducted using standard fluid, CoO NPs and organo-Plg of 0.25 and 0.5 wt %, respectively, as these showed better results in static filtration tests. The tests were performed in duplicate under a hermetically closed system. Figures 9 and 10 present the heating and cooling curves of the studied fluids.

**Figure 9:**
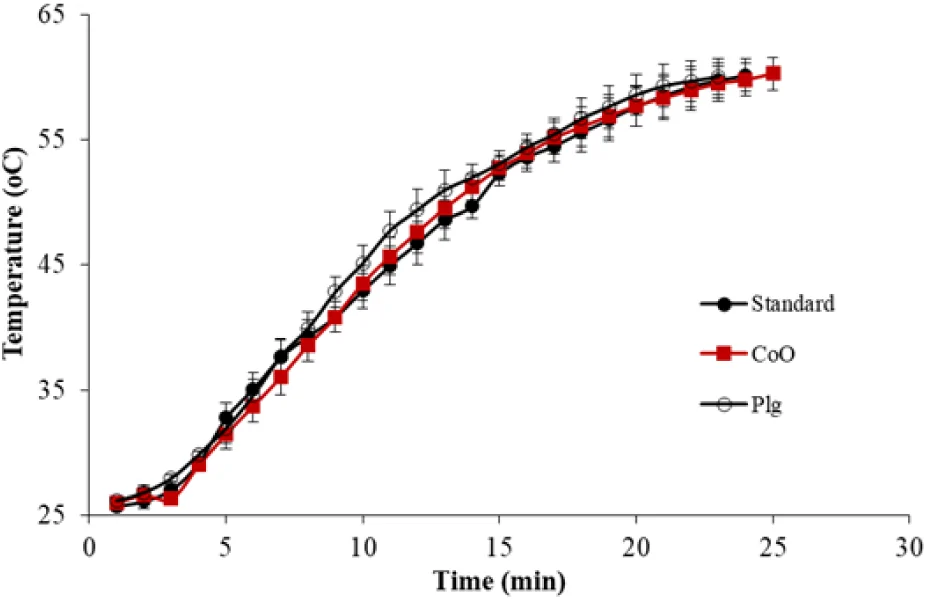
Heating curves of the studied fluids.

**Figure 10:**
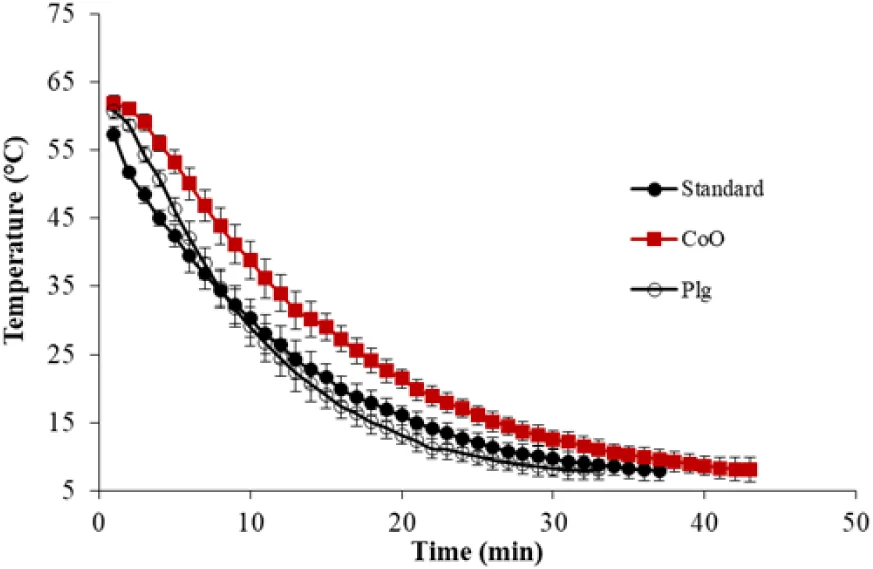
Cooling curves of the studied fluids.

In general, the fluids exhibited similar behavior during heating (Figure 9). However, more pronounced differences were observed in the cooling curves (Figure 10). The fluid containing CoO NPs required a longer time (43 min) to reach thermal equilibrium with the cold environment, indicating greater heat retention compared to the other fluids. The standard fluid and the organo-Plg fluid showed similar behavior; however, the standard fluid reached equilibrium temperature in a longer time (37 min) than the organo-Plg fluid (33 min). By applying Newton’s law of heating and cooling (eqs 5 and 6), the heating (κ_h_) and cooling (κ_c_) coefficients were determined for each fluid, as shown in Table 4.

**Table 4:** Thermal Properties of the Fluids

Fluid	Heating coefficient κ_h_ (min^–1^)	Cooling coefficient κ_c_ (min^–1^)
Standard	0.1697 (±0.02)	0.1326 (±0.01)
CoO	0.1913 (±0.02)	0.1168 (±0.01)
Plg-organo	0.1741 (±0.02)	0.1814 (±0.01)

The κ_h_ values indicate that the CoO NPs fluid (Table 4) exhibits a higher heating rate, which may be associated with an increase in the effective thermal conductivity of the system in the presence of nanoparticles. This behavior is often attributed to the enhanced thermal energy transfer capacity of nanofluids. Upon heating, the thermal motion of the nanofluid may promote better particle dispersion, enhancing heat conduction. However, the lower κ_c_ value for the CoO NPs fluid indicates a slower cooling rate, suggesting greater heat retention capacity. The potential of CoO NPs to improve heat transfer has also been demonstrated by Mitra et al.,^39^ who reported a 21% increase in the thermal conductivity of nanofluids containing 0.011 wt % CoO as the temperature increased from 30 to 60 °C. Since cooling is often dominated by natural convection, it is likely that the nanoparticles helped maintain higher fluid viscosity, reducing convective motion and causing the fluid to lose heat more slowly. This process is facilitated by the thixotropic nature of the fluid across the entire temperature range investigated, as reflected by the GS values. Such behavior may be attributed to attractive interactions between the NPs and other suspended components, particularly polymer chains, which enhance flocculation and contribute to the formation of a three-dimensional network structure.^40^

The fluid containing organo-Plg exhibited a distinct behavior, with higher κ_c_ values than the other fluids, indicating faster heat loss and lower efficiency in low-temperature environments. This is because palygorskite is a fibrous clay with a rod-like structure and high specific surface area (16.5008 m^2^ g^–1^/organo-Plg), higher than that of CoO (4.4341 m^2^ g^–1^), where solid particles act as conductive bridges within the fluid. Organo-Plg behaves as micro-scale heat-conducting channels within the fluid; although these channels may enhance heat transfer during heating, they also facilitate heat dissipation during cooling.^41^

## 4 Conclusion

In this study, the effect of incorporating CoO NPs and organo-Plg on the properties of water-based drilling fluids was evaluated. From a rheological standpoint, the addition of these additives did not promote significant changes. The organo-Plg fluid exhibited stronger electrochemical interactions and, as the CoO NPs fluid, maintained thixotropic behavior with increasing temperature. Regarding the filtration performance, in static filtration tests, the organo-Plg fluid (0.5 wt %) reduced the filtrate volume by 8% at 50 °C. At 70 °C, adsorption of organo-Plg onto PAC LV likely occurred, promoting aggregation and increasing filtrate volume. In the CoO NPs fluid, the filtrate volume increased, possibly due to polymer flocculation induced by the CoO nanoparticles. With respect to the thermal behavior, the CoO NPs fluid exhibited a higher heating coefficient and a lower cooling coefficient, indicating greater heat absorption and retention capacity compared to the other systems. The organo-Plg fluid showed a slight increase (2.6%) in the heating coefficient and a significant increase (36.8%) in the cooling coefficient, suggesting lower thermal retention efficiency. Overall, the results indicate that CoO NPs positively contribute to the thermal properties of the fluid, while organo-Plg shows greater potential for reducing filtrate volume.
